# Interactions Among Nerve Regeneration, Angiogenesis, and the Immune Response Immediately After Sciatic Nerve Crush Injury in Sprague-Dawley Rats

**DOI:** 10.3389/fncel.2021.717209

**Published:** 2021-10-04

**Authors:** Bo He, Vincent Pang, Xiangxia Liu, Shuqia Xu, Yi Zhang, David Djuanda, Guanggeng Wu, Yangbin Xu, Zhaowei Zhu

**Affiliations:** ^1^Department of Orthopaedics, The Third Affiliated Hospital, Sun Yat-sen University, Guangzhou, China; ^2^Department of Plastic Surgery, The First Affiliated Hospital, Sun Yat-sen University, Guangzhou, China; ^3^Department of Plastic Surgery, University of Tennessee Health Science Center, Memphis, TN, United States

**Keywords:** peripheral nerve injury, SD rats, transcriptome, bioinformatic, immune response

## Abstract

To preliminarily explore the primary changes in the expression of genes involved in peripheral nerve processes, namely, regeneration, angiogenesis, and the immune response, and to identify important molecular therapeutic targets, 45 Sprague-Dawley (SD) rats were randomly divided into a control group and an injury group. In the injury group, tissue samples were collected at 4 and 7 days after the injury for next-generation sequencing (NGS) analysis combined with gene ontology (GO) analysis, Kyoto Encyclopedia of Genes and Genomes (KEGG) analysis and Venn diagram construction to identify the differentially expressed mRNAs (DEmRNAs) associated with regeneration, angiogenesis, and the immune response of the nerve. The expression of genes in the distal and proximal ends of the injured nerve after injury was analyzed by qRT-PCR. NGS revealed that compared with the control group, the injury group had 4020 DEmRNAs 4 days after injury and 3278 DEmRNAs 7 days after injury. A bioinformatics analysis showed that C-C chemokine receptor type 5 (CCR5), Thy1 cell surface antigen (Thy1), Notch homolog 1 (Notch1), and semaphorin 4A (Sema4A) were all associated with regeneration, angiogenesis, and the immune response of the nerve at both 4 and 7 days after injury, but qPCR revealed no significant difference in the expression of Thy1 (*P* = 0.29) or Sema4A (*P* = 0.82) in the proximal end, whereas a significant difference was observed in CCR5 and Notch1 (*P* < 0.05). The trend in the Notch1 change was basically consistent with the RNA-seq result after injury, which implied its indispensable role during endothelial cell proliferation and migration, macrophage recruitment, and neurotrophic factor secretion.

## Background

Peripheral nerve regeneration is a complex process. Active Schwann cells (SCs) play an important role in nerve repair process. They not only allow the myelin sheath debris of the axon that disintegrates due to Wallerian degeneration to be cleared quickly but also attract immunocytes migrating to the injured site; this allows the regenerating axons to grow smoothly into the bands of Büngner and eventually improves the efficacy of neurological functional recovery (Bozkurt et al., 2009). We also found in our research that efficacy of peripheral nerve defect repair is related to the blood supply within the graft (Zhu et al., 2017). Vascularized nerve grafting maintains the continuity of the blood supply during nerve transplantation, ensures the activity of SCs, and leads to continuous neurotrophic factor secretion. Due to local ischemia, SCs become necrotic and replaced by fibroblasts, which results in an excessively low concentration of neurotrophic factors secreted by local SCs and slow remyelination of regenerating axons; this is unfavorable for the ingrowth and maturation of regenerating axons (He et al., 2014). Many angiogenic factors are involved in the repair process. Among them, endothelial growth factor (VEGF) and angiopoietin (Ang) are the two most important proangiogenic factors, as they act synergistically in angiogenesis. Studies have shown that VEGF promotes axonal growth through angiogenesis (Ebenezer et al., 2012). In a previous study involving *in vivo* experiments in rats, we found that COMP-Ang1, an angiogenic factor, was able to promote early angiogenesis in nerve repair scaffolds and the proliferation of SCs in peripheral nerves (Qiu et al., 2015). Besides, immune response provided significant assistance, especially during the early phrase after nerve injury. The myelin sheath debris is phagocytosed and removed by macrophages, which are brought in by the blood supply, and local SCs. At the same time, immune cells also secrete some cytokines that promote proximal nerve fiber growth to form growth cones (Lindborg et al., 2018).

Although axonogenesis, angiogenesis and the immune response are three different processes, they have numerous interactions after nerve injury (Cattin et al., 2015). Many cytokines and genes are involved in the mutual regulation of regeneration, angiogenesis, and the immune response in the peripheral nerve, but the specific mechanism remains unclear. To further study the molecular mechanisms of regeneration, angiogenesis, and the immune response in the nerve after peripheral nerve injury, this study aimed to perform transcriptome analysis of injured nerves in a rat model of sciatic nerve crush injury induced by clamping. We preliminarily explored the primary changes in gene expression that are involved in regeneration, angiogenesis, and the immune response of the peripheral nerve and provide a scientific basis for more effective treatment of peripheral nerve injury by identifying important molecular therapeutic targets.

## Materials and Methods

### Animal Selection and Sample Collection

**FIGURE 1:**
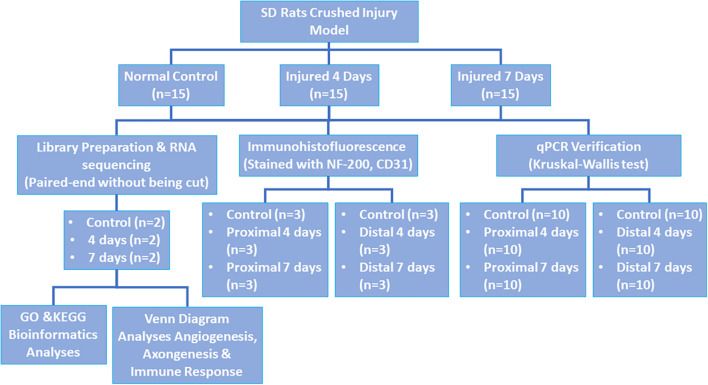
Study design.

Forty-Five healthy male Sprague-Dawley (SD) rats weighing approximately 150–200 g were chosen and randomly separated into three groups as follows: control, 4-day injury, and 7-day injury groups (15 rats per group). This study was approved by the Experimental Animal Administration Committee of Sun Yat-sen University. Efforts were taken to minimize animal suffering during the experiment. The study design is presented in Figure 1.

### Sciatic Nerve Damage Model and Sampling

Experimental rats (injured for 4 days and injured for 7 days) were anesthetized by intraperitoneal injection of 10% chloral hydrate (0.3 ml/100 g) (Sinopharm Chemical Reagent Co., Ltd., Shanghai, China) and were subjected to sciatic nerve crush injury according to a previously published procedure (Zhu et al., 2017). Under aseptic conditions, the skin of the left leg was cut parallel to the femur, and the sciatic nerve was exposed by splitting the superficial gluteus muscle. The middle segment of the left sciatic nerve was clamped with hemostatic forceps for approximately 30 s. Subsequently, they were released, and the wound was closed, after which the animals were kept in a quiet and comfortable environment with a temperature between 20 and 25°C and humidity between 50 and 65%, and enough food and water were ensured. We also offered light for 14 h to maintain the normal circadian rhythm of each animal. The condition of the animals and the wounds were observed regularly.

### Immunofluorescence

Three rats were randomly selected from each group. In the 4-day and 7-day injury groups, the nerve was completely cut at the lesion site at each selected observation time, and samples were obtained 1 cm proximal to the lesion and 1 cm distal to the lesion. The nerve samples were fixed in 4% paraformaldehyde at 4°C for 24 h. Subsequently, the nerve was placed in 20% sucrose for 24 h for the first round of dehydration, which was followed by placement in 30% sucrose for 24 h for the second round of dehydration. Ten-micrometer-thick sections of the middle segment of the nerve were cut using a cryostat and placed on a coverslip; immunofluorescence staining was then performed with a rabbit anti-mouse NF-200 antibody (Sigma-Aldrich, Tokyo, Japan, 1:400), a CD31 antibody (Sigma-Aldrich, Tokyo, Japan, 1:400), and a FITC-conjugated goat anti-rabbit IgG secondary antibody (TCS Biologicals, Botolph Clayton, United Kingdom; 1:200). Antibodies were diluted in PBS containing 3% rat serum, 3% goat serum, and 0.02% sodium azide (Sigma-Aldrich, Tokyo, Japan) to reduce background staining.

For image study, photos were taken under the same condition as previously described (Zhu et al., 2015). For the determination of NF200 and CD31 expression, the integrated optical density (IOD) of positive staining was assessed using Image-Pro Plus. For each animal and for each stain, calculations were made on three randomly chosen sections, and the results were averaged.

### Library Preparation and RNA Sequencing

Two rats were randomly selected from each group, and the injured nerves were harvested as samples for next-generation RNA sequencing (NGS). All sequencing programs were performed by Shanghai Biotechnology Corporation (Shanghai, China).

During transcriptome sequencing, to enable comparability of gene expression levels among different genes and different samples, the reads were converted into FPKM (fragments per kilobase of transcript per million mapped reads) to normalize the gene expression level (Mortazavi et al., 2008). We first applied Stringtie (version: 1.3.0) (Pertea et al., 2015; Pertea et al., 2016) to count the number of fragments for each gene after HisAT2 alignment, and then we used the TMM (trimmed mean of *M* values) (Robinson and Oshlack, 2010) method for normalization; finally, we used Perl script to calculate the FPKM value for each gene.

### Bioinformatics Analyses

EdgeR (Robinson et al., 2010) was used to perform the differential gene analysis between samples. After the *P*-value was derived, multiple hypothesis testing correction was performed, and the threshold of the *p*-value was determined by controlling the FDR (false discovery rate) (Benjamini et al., 2001); the corrected *p*-value is the *q*-value. Moreover, we calculated the fold differential expression based on the FPKM values, namely, the fold change. Differential gene screening conditions were set at a *q*-value ≤ 0.05 and a fold change ≥ 2. Both gene ontology (GO) analysis^1^ and KEGG pathway analysis^2^ were performed to determine the roles of the differentially expressed mRNAs. Fisher’s exact test was used to test the significance of GO terms and KEGG pathway identifier enrichment in the differentially expressed gene list. RNA sequencing and bioinformatics analyses were carried out by Shanghai Biotechnology Corporation (Shanghai, China).

We defined axonogenesis, angiogenesis, and immune response by GO categories, whose item numbers were 0007409, 0001525, and 0006955, respectively. The upregulated differentially expressed mRNAs (DEmRNAs) related to these three items were analyzed, and a Venn diagram was generated by web tools^3^.

### Quantitative Real-Time RT-PCR

Ten rats were randomly selected from each group. In the 4-day and 7-day injury groups, the nerve was completely transected at the lesion site, and samples were obtained 1 cm proximal to the lesion and 1 cm distal to the lesion. Quantitative analysis of the targeted mRNA expression was performed using real-time RT-PCR as previously described (Djuanda et al., 2021). The RNA concentration and purity were detected using a NanoDrop 2000, and RNA with an excessively high concentration was diluted appropriately to a final concentration of 200 ng/μl. One microgram of each specimen was used for RNA reverse transcription using the RevertAid First Strand cDNA Synthesis Kit (Thermo, MA, United States). An appropriate amount of cDNA was amplified using FastStart Universal SYBR Green Master Mix (Roche, Basel, Switzerland) in a fluorescence quantitative PCR machine (StepOne Plus, Thermo, MA, United States). For the specific procedure, please refer to the product manual. This experiment was repeated three times for each specimen. GAPDH expression was used to normalize the expression of mRNAs, and information about the genes to be tested and their primers is shown in Table 1. The specificity of real-time PCR was confirmed via routine agarose gel electrophoresis and melting-curve analysis. The 2^–ΔΔ^ ^Ct^ method was used to calculate relative gene expression. GAPDH served as the reference gene.

**TABLE 1:** List of qRT-PCR primers.

Gene name	Sequence
GAPDH	Forward: 5′-TTCCTACCCCCAATGTATCCG-3′ Reverse: 5′-CATGAGGTCCACCACCCTGTT-3′
CCR5	Forward: 5′-CCTAAATCACTGAGGCGGTCAG-3′ Reverse: 5′-TGGCCACTTACCACAGAGCTA-3′
Thy1	Forward: 5′-CATGACCAGCTCGCAGACCTA-3′ Reverse: 5′-CTGGAGTGGGAGGAAGAGGTAA-3′
Notch1	Forward: 5′-TGTCGCTGGGTACAAATGCAA-3′ Reverse: 5′-A CGGTAGCTGCCATTGGTGTTC-3′
Sema4A	Forward: 5′-TGTCGCTGGGTACAAATGCAA-3′ Reverse: 5′-A CGGTAGCTGCCATTGGTGTTC-3′

### Gene Interaction Analysis

Network software built by Cytoscape (version: 3.7.1^4^) with the GeneMania plug-in (version: 3.5.1^5^) was used to analyze the interactions between genes sorted by the Venn diagram. *Rattus norvegicus* was chosen, and the parameter was set to the top 20 related genes using automatic weight. Through the score ranking of each gene, the network of genes interacting with the sorted genes was obtained.

### Statistical Analysis

The RT-PCR data are presented as the mean ± SD as describedpreviously (Liu et al., 2018). SPSS 16.0 software (SPSS, Chicago, IL, United States) was used to analyze quantitative RT-PCR results. Statistical analysis was performed with Kruskal–Wallis test to determine the significant differences between the groups. α < 0.05 was considered statistically significant.

## Results

### Staining of Nerve and Blood Vessel Markers in the Sciatic Nerve of Sprague-Dawley Rats After Injury

**FIGURE 2:**
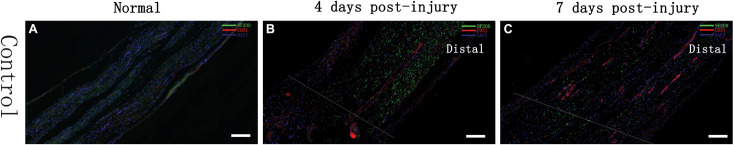
Immunofluorescence assessment of the sciatic nerve in SD rats Changes in the expression of an axon-associated protein (NF-200) and a vascular endothelial cell protein (CD31) in the early stage of a contusion injury (4 days, 7 days) compared with controls. The scale bar represents 200 μm. **(A)** Normal rat sciatic nerve; **(B)** 4 days after injury, rat sciatic nerve injury and beyond; **(C)** 7 days after injury, rat sciatic nerve injury and beyond.

The sciatic nerves of SD rats in the control group and those of SD rats at 4 and 7 days after injury were obtained to examine the expression of the nerve axon marker NF-200 and the vascular endothelial cell marker CD31. The results showed that NF200 expression was negatively correlated with time and that its expression gradually changed from a line-like distribution to a punctate scattered distribution, as observed by microscopy; however, CD31 expression was positively correlated with time, and multiple small luminal structures were observed inside the nerve at day 7 (see Figure 2). Additionally, we found that compared to control group, the injury group had a decrease in NF200-positive fibers after injury, and on day 7, most NF200-positive staining had disappeared. These phenomena suggested that Wallerian degeneration of sciatic nerves after injury was ongoing and that our crush models were established successfully. In contrast to the transection models, the crush models still had some NF200 + axon fibers after injury, which were evident by immunofluorescence staining at day 7.

Analysis of IOD demonstrated that the expression of NF200 was higher in control group (12262 ± 2863), and decreased gradually after injury in day 4 (7478 ± 1603) and day 7 (4061 ± 2933). Same trend appeared in CD31 expression, which showed highest in control group (12043 ± 2849), then decrease in day 4 (1374 ± 848) and day 7 (3595 ± 2011).

### Transcriptome Analysis After Clamp-Induced Sciatic Nerve Crush Injury in Sprague-Dawley Rats

**FIGURE 3:**
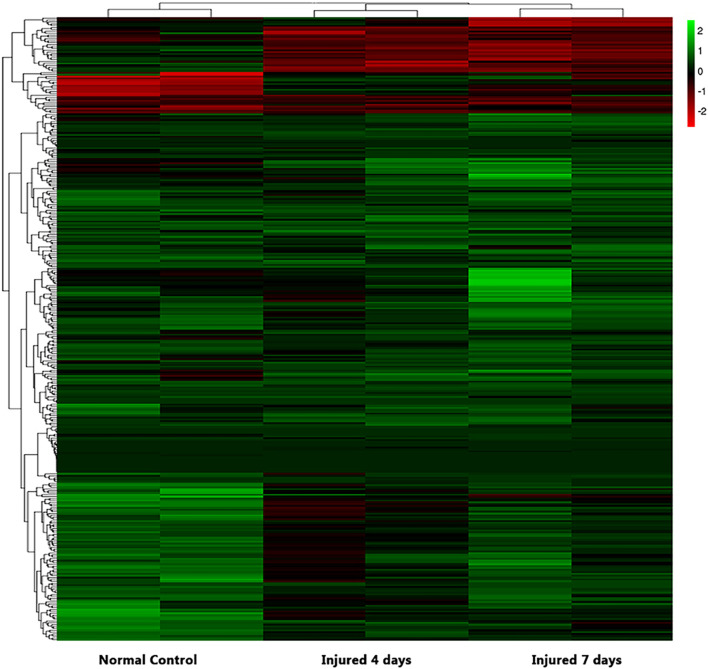
Heatmap of differential mRNA expression after RNA sequencing at different times (4 and 7 days after injury) soon after sciatic nerve contusion in SD rats. Black indicates no significant fold change in mRNA expression between the groups, green indicates increased expression, and red indicates decreased expression.

We used NGS to analyze the expression of genes in the transcriptome at 4 and 7 days after clamp-induced sciatic nerve crush injury in SD rats, and the distribution of differentially expressed mRNAs (DEmRNAs) is shown in heat maps (Figure 3). In all, 30,957 mRNAs were detected by RNA-seq and were screened according to the criteria of a fold change ≥ 2.0 and *q* < 0.05. Compared with the control rats, rats examined 4 days after clamp-induced crush injury had 4020 DEmRNAs, with the expression of 2500 being upregulated and 1520 being downregulated. Four days after injury, 137 mRNAs were activated in the experimental group, and of these, the most obvious change was in Hmga2 (FPKM = 0 vs. 16.42, *q* < 0.05). Sixty-four genes were silenced, and the greatest change was seen in fgf4 (FPKM = 4.00 vs. 0, *q* < 0.05). The top 10 DEmRNAs that were activated or silenced are shown in Table 2. In addition, among the mRNAs that were expressed in both the control and experimental groups, the mRNA with the most significant increase was Tyrp1 (log2FC = 9.75, *q* < 0.05), whereas the one with the largest decrease was Sec14l5 (log2FC = −11.16, *q* < 0.05). The top 10 DEmRNAs that were the most significantly upregulated or downregulated are shown in Table 3. Seven days after injury, there were 3278 DEmRNAs; expression of 1977 mRNAs was upregulated, and expression of 1301 mRNAs were downregulated. Among them, 88 mRNAs were activated, and the most obvious change was seen in Apoc1 (FPKM = 0 vs. 5.96, *P* < 0.05); 35 mRNAs were silenced, and the most obvious change was seen in Hapln2 (FPKM = 4.26 vs. 0, *P* < 0.05). The top 10 DEmRNAs that were activated or silenced are shown in Table 4. In addition, among the mRNAs that were expressed in both the control and the experimental groups, the mRNA with the largest increase was tyrosinase-related protein 1 (Tyrp1) (log2FC = 10.92, *P* < 0.05), whereas the one with the largest decrease was Rn60_10_0890.5 (log2FC = −8.63, *P* < 0.05). The top 10 DEmRNAs that were the most significantly upregulated or downregulated are shown in Table 5.

**TABLE 2:** Top 10 DEmRNAs only expressed 4 days after crush injury (Post-Injury 4 days, PI-4 d) or only expressed in normal sciatic nerves.

	Gene name	Control	PI-4d	*P*-value	*q*-value
Only in PI-4d	Hmga2	0	16.42365	1.10E-40	9.60E-39
	Draxin	0	4.227762	6.20E-21	1.79E-19
	Pth2r	0	2.709091	1.31E-22	4.29E-21
	Podnl1	0	2.029091	9.10E-17	1.94E-15
	U4	0	1.946587	0.008691	0.028003
	AABR07030791.1	0	1.931831	1.98E-10	2.41E-09
	SNORA26	0	1.701125	0.013095	0.039665
	Apoc1	0	1.623794	5.68E-09	5.87E-08
	Mmp7	0	1.524575	2.90E-05	0.000173
	Mar11	0	1.373298	6.41E-13	9.82E-12
Only in control group	Fgf4	3.996402849	0	7.56E-16	1.49E-14
	SNORA24	3.248064983	0	0.000219535	0.001086115
	C1qtnf4	2.995813298	0	3.86E-13	6.03E-12
	SNORA17	2.137505294	0	0.003996423	0.014321861
	7SK	1.846016982	0	7.86E-06	5.18E-05
	Mobp	1.845934814	0	6.50E-21	1.87E-19
	Omg	1.460558332	0	1.71E-16	3.57E-15
	AABR07030053.1	1.124891401	0	0.000367528	0.001730692
	Fam163b	1.039812773	0	4.98E-15	9.22E-14
	AABR07043929.1	1.029593343	0	0.003062435	0.011285335

**TABLE 3:** Top 10 up/down regulated DEmRNAs 4 days after crush injury.

	Gene name	Control	PI-4d	log2FC	*P*-value	q-value
Up	Tyrp1	0.044697	38.389	9.74631	7.74E-151	2.88E-147
	Tnn	0.006946	4.546313	9.35422	2.66E-24	9.86E-23
	Mmp12	0.014896	4.753194	8.31787	3.46E-11	4.59E-10
	Gpr83	0.102426	30.33552	8.21029	4.74E-99	3.39E-96
	Ucn2	0.135147	36.98082	8.0961	4.40E-75	1.34E-72
	Gdnf	0.171444	36.09137	7.71778	2.13E-57	3.89E-55
	Mrap2	0.010181	2.045476	7.65046	1.04E-17	2.38E-16
	Fam132b	0.021671	4.319474	7.63894	2.13E-19	5.65E-18
	Fgf5	0.222784	39.20535	7.45926	3.66E-62	7.74E-60
	Cabp2	0.016972	2.560325	7.23702	2.74E-18	6.54E-17
Down	Sec14l5	20.16116	0.00883	–11.15691	1.45E-111	1.35E-108
	B3galt2	6.578876	0.004418	–10.54028	1.59E-77	5.49E-75
	LOC688613	13.08011	0.023957	–9.092689	2.49E-40	2.13E-38
	4932411E22Rik	16.50995	0.032553	–8.986331	7.87E-54	1.25E-51
	Lect1	189.2592	0.667838	–8.14665	1.18E-141	3.65E-138
	Ncmap	560.4528	2.046832	–8.097057	4.11E-140	1.09E-136
	Slco4c1	1.55139	0.008964	–7.435194	6.11E-31	3.26E-29
	Dcdc2	3.533837	0.020657	–7.418444	2.33E-67	5.71E-65
	Slc9a3	18.51888	0.116726	–7.309727	4.92E-128	8.30E-125
	Asb15	1.636855	0.010325	–7.308676	1.15E-27	5.15E-26

**TABLE 4:** Top 10 DEmRNAs only expressed 7 days after crush injury (Post-Injury 7 days, PI-7d) or only expressed in normal sciatic nerves.

	Gene name	Control	PI-7d	*P*-value	*q*-value
Only in PI-7d	Apoc1	0	5.970197	4.70E-17	2.95E-15
	Apoc4	0	5.188777	1.76E-13	6.82E-12
	AABR07030791.1	0	4.193796	1.22E-17	8.19E-16
	Hmga2	0	4.171533	4.29E-14	1.78E-12
	Pth2r	0	2.709295	2.96E-20	2.65E-18
	SNORA17	0	2.637213	0.004973	0.024347
	Mmp7	0	2.629549	5.82E-11	1.64E-09
	LOC683295	0	2.378256	7.81E-15	3.53E-13
	Draxin	0	1.958597	2.17E-11	6.48E-10
	Pnlip	0	1.601258	3.65E-11	1.06E-09
Only in normal nerves	Hapln2	4.256182914	0	1.07E-21	1.13E-19
	AABR07034833.1	3.00758614	0	3.20E-07	4.82E-06
	RGD1309651	1.150919443	0	0.002781747	0.014995912
	AABR07043929.1	1.029593343	0	0.003365706	0.017661869
	AABR07069211.1	0.971437254	0	0.000191145	0.001516581
	AABR07044148.1	0.743679909	0	0.000472723	0.003320686
	AABR07059527.1	0.647496594	0	0.005470721	0.026331135
	AABR07061178.1	0.611046904	0	0.000170623	0.001376103
	AABR07042654.1	0.595353922	0	0.006749527	0.03130678
	Htr3b	0.554774052	0	9.43E-06	0.000105647

**TABLE 5:** Top 10 up/down regulated DEmRNAs 7 days after crush injury.

	Gene name	Control	PI-7d	log2FC	*P*-value	*q*-value
Up	Tyrp1	0.044697	86.81279	10.9235	1.40E-103	2.61E-99
	Gpr83	0.102426	62.51478	9.25348	3.13E-70	1.16E-66
	Mmp12	0.014896	3.421537	7.84362	7.88E-18	5.36E-16
	Mrap2	0.010181	1.772636	7.44392	1.35E-14	5.92E-13
	Tnn	0.006946	1.147016	7.36741	3.74E-19	2.95E-17
	Mmp8	0.038257	5.635753	7.20274	1.44E-21	1.49E-19
	Ucn2	0.135147	19.90056	7.20214	8.34E-37	4.69E-34
	Fgf5	0.222784	27.30074	6.93715	5.94E-40	4.80E-37
	Cabp2	0.016972	1.920472	6.82216	1.78E-13	6.88E-12
	Tfap2c	0.024944	2.734148	6.77627	5.83E-17	3.61E-15
Down	Rn60_10_0890.5	6.734551	0.017053	–8.625381	7.44E-05	0.000669
	Myh7	1.921069	0.025591	–6.23013	3.68E-25	6.57E-23
	Krt5	0.485026	0.006569	–6.206171	8.18E-10	1.91E-08
	Slco4c1	1.55139	0.021535	–6.170702	4.23E-24	6.05E-22
	Ninj2	0.921764	0.016422	–5.810661	6.83E-09	1.39E-07
	C1qtnf4	2.995813	0.054888	–5.770303	2.65E-09	5.68E-08
	Fgf4	3.996403	0.085413	–5.548109	1.21E-11	3.73E-10
	Slc46a2	0.517902	0.013404	–5.271998	4.39E-07	6.42E-06
	Fam163b	1.039813	0.027344	–5.248954	3.61E-11	1.05E-09
	4932411E22Rik	16.50995	0.466168	–5.146343	2.05E-32	8.08E-30

### Gene Ontology Analysis and Kyoto Encyclopedia of Genes and Genomes Analysis of DEmRNAs

**FIGURE 4:**
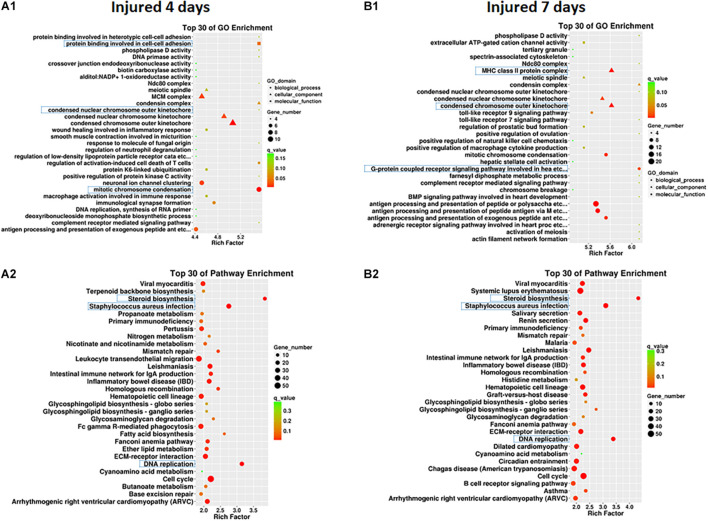
GO enrichment map **(A1,B1)** and KEGG pathway enrichment map **(A2,B2)** of differentially expressed mRNAs in SD rats 4 and 7 days after sciatic nerve injury compared with rats without injury during the early stage after sciatic nerve contusion. **(A1)** GO gene enrichment map at 4 days after injury; **(B1)** GO gene enrichment map at 7 days after injury. The different shapes in the figure represent different categories, with circles representing biological processes, triangles representing cellular components, and squares representing molecular functions. A larger shape area indicates a greater number of DEmRNAs in that category. The significant differences in the distribution of DEmRNAs in different subcategories between the two groups are indicated by different colors, where green indicates *q*-values > 0.15, yellow indicates that *q*-values are between 0.10 and 0.15, orange indicates that *q*-values are between 0.05 and 0.10, and red indicates *q*-values < 0.05. The test criterion was set at 0.05. **(A2)** KEGG gene enrichment map at 4 days after injury; **(B2)** KEGG gene enrichment map at 7 days after injury. A larger graphical area in the figure indicates a higher number of DEmRNAs in that channel. The significant differences in the distribution of DEmRNAs in different channels between the two groups are indicated by different colors, where green indicates *q*-values > 0.3, yellow indicates that *q*-values are between 0.2 and 0.3, orange indicates that *q*-values are between 0.1 and 0.2, and red indicates *q*-values < 0.1. The test criterion was 0.1.

Clustering analysis of DEmRNAs can help to identify the function of unknown transcripts or the unknown function of known transcripts by gathering similar expression patterns or similar genes to a class (Yao et al., 2012). The results showed that at 4 days after injury, DEmRNAs were enriched mainly in mitotic chromosome condensation [Rich factor (RF) = 5.52, *q* < 0.05], protein binding involved in cell-cell adhesion (RF = 5.52, *q* < 0.05), and condensed chromosome outer kinetochore (RF = 5.06, *q* < 0.05). However, 7 days after injury, DEmRNAs were enriched mainly in the G protein-coupled receptor signaling pathway involved in heart processes (RF = 6.14, *q* < 0.05), condensed chromosome outer kinetochores (RF = 5.62, *q* < 0.05), and MHC class II protein complexes (RF = 5.62, *q* < 0.05). The GO enrichment diagram and the distribution of GO entries enriched in the top 10 are shown in Figures 4A1,B1 and Table 6.

**TABLE 6:** The classification corresponding to the top 10 DEmRNAs at 4 and 7 days after sciatic nerve crush injury in SD rats in the GO analysis.

	GO_ID	GO_term	Rich factor	*P*-value	*q*-value
4 Days	GO:0007076	Mitotic chromosome condensation	5.522492	0.000172	0.004143
	GO:0098632	Protein binding involved in cell–cell adhesion	5.522492	0.002896	0.040288
	GO:0000940	Condensed chromosome outer kinetochore	5.062285	0.000148	0.003641
	GO:0000778	Condensed nuclear chromosome kinetochore	4.908882	0.001208	0.020329
	GO:0042555	MCM complex	4.518403	0.000984	0.017223
	GO:0045161	Neuronal ion channel clustering	4.518403	0.000984	0.017223
	GO:0019886	Antigen processing and presentation of exogenous peptide antigen via MHC class II	4.417994	0.001983	0.029688
	GO:0006270	DNA replication initiation	4.417994	2.03E-05	0.000666
	GO:0002504	Antigen processing and presentation of peptide or polysaccharide antigen via MHC class II	4.321951	8.20E-06	0.000304
	GO:0042613	MHC class II protein complex	4.141869	0.001556	0.02449
7 Days	GO:0086103	G-protein coupled receptor signaling pathway involved in heart process	6.136293	0.000758	0.014837
	GO:0000940	Condensed chromosome outer kinetochore	5.624935	5.94E-05	0.001758
	GO:0042613	MHC class II protein complex	5.624935	5.94E-05	0.001758
	GO:0019886	Antigen processing and presentation of exogenous peptide antigen via MHC class II	5.522664	0.000278	0.006433
	GO:0007076	Mitotic chromosome condensation	5.522664	0.000278	0.006433
	GO:0000778	Condensed nuclear chromosome kinetochore	5.454483	0.000607	0.012311
	GO:0002495	Antigen processing and presentation of peptide antigen via MHC class II	5.369256	1.01E-05	0.000372
	GO:0002504	Antigen processing and presentation of peptide or polysaccharide antigen via MHC class II	5.335907	1.79E-07	1.18E-05
	GO:0034162	Toll-like receptor 9 signaling pathway	5.25968	0.002935	0.043712
	GO:0008331	High voltage-gated calcium channel activity	4.772672	0.002204	0.03514

Pathway enrichments was performed by means of KEGG analysis to determine the pathways in which DEmRNAs are involved during the early stage after injury to understand the molecular interactions after injury. The results showed that the top three pathways enriched were steroid biosynthesis (3.84 vs. 4.33, *q* < 0.05), DNA replication (3.15 vs. 3.39, *q* < 0.05), and *Staphylococcus aureus* infection (2.75 vs. 3.11, *q* < 0.05) at both 4 and 7 days after injury. The KEGG enrichment diagram and the distribution of the top 10 GO entries enriched are shown in Figures 4A2,B2 and Table 7.

**TABLE 7:** The classification corresponding to the top 10 DEmRNAs at 4 and 7 days after sciatic nerve crush injury in SD rats in the KEGG analysis.

	Pathway_ID	Pathway description	Rich factor	*P*-value	*q*-value
4 Days	rno00100	Steroid biosynthesis	3.841198	7.69E-06	0.000448
	rno03030	DNA replication	3.148276	2.90E-06	0.000281
	rno05150	*Staphylococcus aureus* infection	2.754066	1.12E-06	0.000163
	rno03430	Mismatch repair	2.432759	0.005113	0.035425
	rno03440	Homologous recombination	2.432759	0.002673	0.025091
	rno04672	Intestinal immune network for IgA production	2.211599	0.000987	0.013053
	rno04110	Cell cycle	2.211599	1.59E-07	4.61E-05
	rno05140	Leishmaniasis	2.185957	7.34E-05	0.003562
	rno05321	Inflammatory bowel disease (IBD)	2.162452	0.000184	0.005962
	rno03460	Fanconi anemia pathway	2.115442	0.001708	0.017138
	rno00100	Steroid biosynthesis	4.331055	1.72E-06	9.89E-05
7 Days	rno03030	DNA replication	3.388414	1.47E-06	0.000141
	rno05150	*Staphylococcus aureus* infection	3.105285	9.14E-08	2.63E-05
	rno00604	Glycosphingolipid biosynthesis – ganglio series	2.743002	0.009715	0.049089
	rno05140	Leishmaniasis	2.464726	7.54E-06	0.00031
	rno04924	Renin secretion	2.351144	5.96E-05	0.001561
	rno05310	Asthma	2.351144	0.004946	0.031656
	rno05332	Graft-versus-host disease	2.334469	0.000499	0.006537
	rno03440	Homologous recombination	2.321001	0.007416	0.043586
	rno04110	Cell cycle	2.266943	2.71E-07	3.91E-05

### Triple Intersection of Nerve Regeneration, Angiogenesis and Immune Response Among Upregulated DEmRNAs at 4 and 7 Days After Injury and Validation by qPCR

At 4 days after injury, 6 DEmRNAs were found to be involved in both nerve regeneration and angiogenesis: semaphorin 3E (Sema3e), fibroblast growth factor 8 (Fgf8), neural cell adhesion molecule (Ncam), ephrin type-B receptor 3 (Ephb3), Sonic hedgehog (Shh), and TNF receptor superfamily member 12A (Tnfrsf12a). At 7 days after injury, increased expression in only Sema3e, Ephb3, Shh, and Tnfrsf12a was maintained (*P* < 0.05). At 4 and 7 days after injury, 2 DEmRNAs were found to be involved in both regeneration and the immune response of the nerve: nerve growth factor receptor (Ngfr) and Sema7a (*P* < 0.05).

**FIGURE 5:**
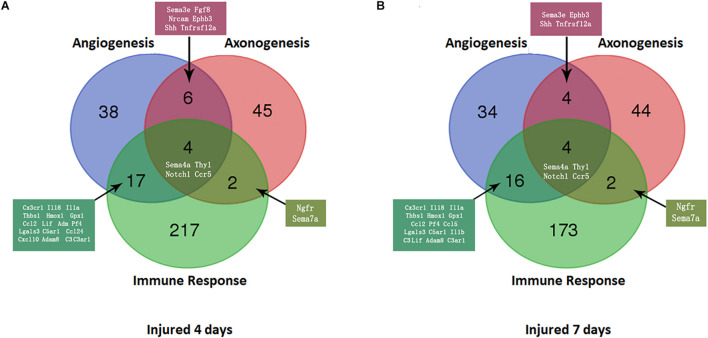
Venn diagram of mRNAs with increased expression at the intersection of angiogenesis, axonal regeneration, and the immune response at different time points. **(A)** 4 days after sciatic nerve crush injury; **(B)** 7 days after sciatic nerve crush injury. The number and names of intersecting genes are indicated in the figure.

A Venn diagram was used to perform the three-intersection analysis of the DEmRNA-associated signaling pathways of regeneration, angiogenesis, and the immune response of the nerve, and the intersection of the upregulated DEmRNAs at 4 and 7 days after injury is shown in Figure 5. The results showed that the intersecting mRNAs of regeneration, angiogenesis, and the immune response of the nerve at 4 and 7 days after injury included C-C chemokine receptor type 5 (Ccr5), Thy1 cell surface antigen (Thy1), Notch homolog 1 (Notch1), and Sema4A, and the expression levels of all but Thy1 were significantly increased compared with the control group (*P* < 0.05).

**FIGURE 6:**
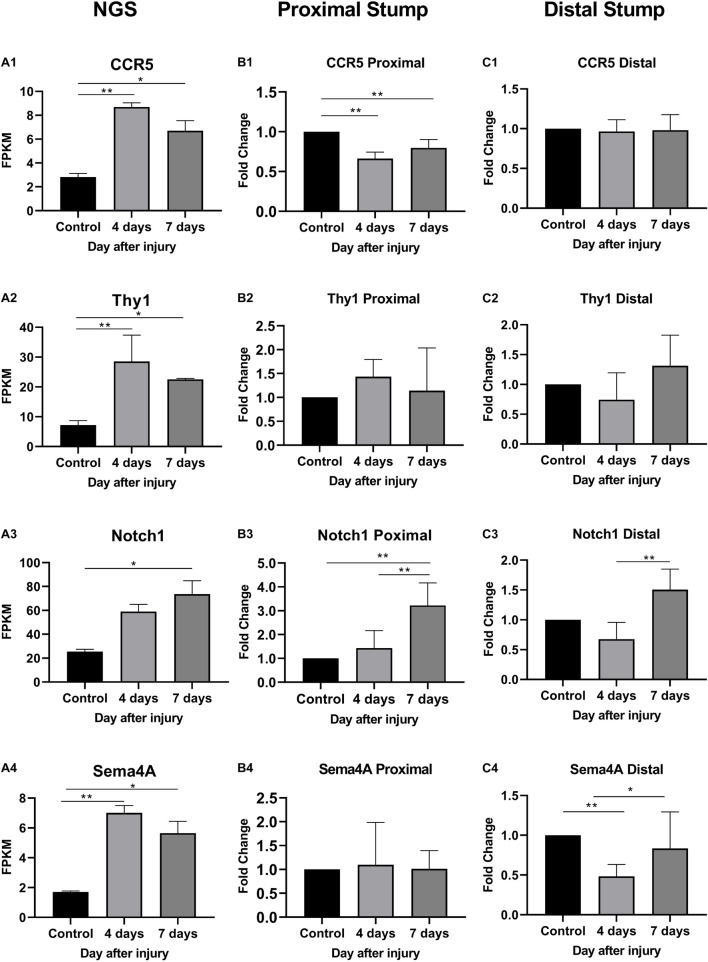
Expression levels of DEmRNAs common to angiogenesis, axonal regeneration and immune response. **(A)** NGS results; **(B)** qRT-PCR expression of genes in proximal part after injury; **(C)** qRT-PCR expression of genes in distal part after injury. **(A1–C1)** CCR5 expression; **(A2–C2)** Thy1 expression; **(A3–C3)** Notch1 expression; **(A4–C4)** Sema4a expression. * denotes *P* < 0.05 compared with the control group, and ** denotes *P* < 0.01 compared with the control group.

As shown in Figure 6, In the proximal ends of the injured nerve between the experimental group and the control group, the qPCR results revealed no significant difference in the expression of Thy1 (*P* > 0.05) or Sema4A (*P* > 0.05), whereas a significant difference was observed in CCR5 and Notch1 (*P* < 0.05); however, the trends in CCR5 was not completely consistent with the RNA-seq results. At 4 days after injury, the expression of CCR5 was significantly reduced (FC = 0.66 ± 0.02, *P* < 0.01), gradually increased and then remained lower than that of the control group at day 7 (FC = 0.80 ± 0.03, *P* < 0.01). The trend in the Notch1 change was basically consistent with the RNA-seq result after injury. In the proximal ends of injured nerves, Notch1 expression showed significant changes 4 days after the operation and continued to increase at day 7.

In distal ends of the injured nerves, the results of the three-intersection analyses in the control group, the 4-day injury group, and the 7-day injury group showed no significant difference in the expression of CCR5 (*P* > 0.05), but significant differences were observed in the expression of Thy1, Notch1, and Sema4A between the groups (*P* < 0.05). Compared with that in the control group, the expression of Thy1 was not significantly different in the day-4 (FC = 0.74 ± 0.16) or day-7 injury group (FC = 1.31 ± 0.18, *P* > 0.05), but Thy1 expression was significantly higher at day 7 than at day 4 (*P* < 0.05). Four days after injury, Notch1 expression was significantly lower than that in the control group (FC = 0.68 ± 0.10, *P* < 0.01); however, Notch1 expression then gradually increased and became significantly higher than that in the control group by day 7 (*P* < 0.01). Four days after injury, Sema4A expression was significantly reduced compared with that in the control group (FC = 0.48 ± 0.05, *P* < 0.01), and its expression gradually increased thereafter (FC = 0.86 ± 0.16, *P* > 0.05).

### Analysis of the Intersecting Genes and Their Interactions With Related Genes

**FIGURE 7:**
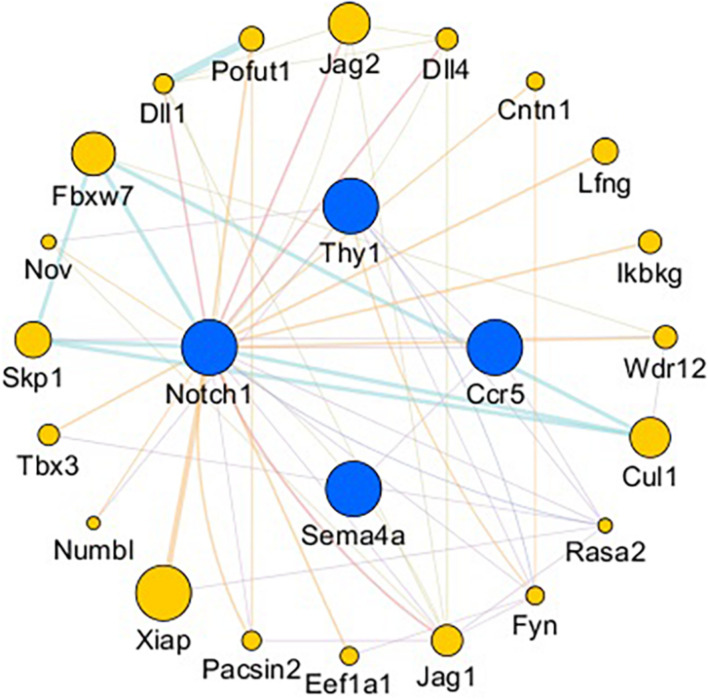
Gene interaction network. Blue nodes: Genes related to axonogenesis, angiogenesis, and the immune response. Yellow nodes: Genes that interacted with CCR5, Thy1, Notch1, and Sema4A. The node size is based on the score. The larger the node is, the higher the score will be.

The gene network of the mutual intersections among the 21 genes with the highest correlation with Ccr5, Thy1, Notch1, and Sema4A is shown in Figure 7 and Supplementary Tables 1, 2. The results revealed a total of 47 links, of which co-expression accounted for 42.55%, predicted links accounted for 29.79%, and physical interactions accounted for 8.51%. Higher scores of the nodes indicate better correlation with the screened genes. The three nodes with the highest scores were X-linked inhibitor of apoptosis (Xiap, score: 0.012), F-box and WD repeat domain containing 7 (Fbxw7, score: 0.009) and Jagged 2 (Jag2, score: 0.008), and 8 were associated with the Notch signaling pathway.

## Discussion

**FIGURE 8:**
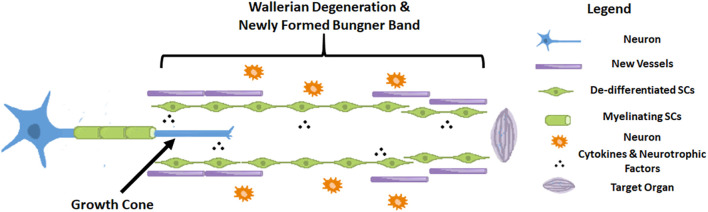
Schematic diagram of a nerve after injury.

Axonogenesis, angiogenesis and the immune response have numerous interactions after nerve injury (Cattin et al., 2015). In order to better verified cell clusters involved in the nerve injury and repair process, Kalinski et al. (2020) performed single-cell RNA sequencing in mice sciatic nerves after crush injury models. After being injured for 3 days, 24 different cell clusters were identified. The most prominently featured are immune cells, especially innate immune cells (Itgam/CD11b^+^). Other abundantly featured cell types include mesenchymal progenitor cells, Schwann cells and nerve vasculature cells. The early pathophysiological changes after peripheral nerve injury are reflected in Wallerian degeneration of the distal end and part of the proximal end of the nerve. The myelin sheath debris is phagocytosed and removed by macrophages, which are brought in by the blood supply, and local SCs. As we found in our research that at the very beginning after injury, NF-200 positive fibers were fragmented and finally disappeared, while CD31 positive vasculature was immediately established. At the same time, cells also secrete some cytokines that promote proximal nerve fiber growth to form growth cones. Only when the proximal regenerating axons pass through the site of injury, regenerate distally, and innervate the target organ is the process of nerve regeneration ultimately completed (Sayad Fathi and Zaminy, 2017). During this process, a series of signaling pathways regulate the local microenvironment inside the nerve until nerve regeneration is completed (Kato et al., 2013; Liu et al., 2015, 2016; Figure 8).

In the adult mammalian peripheral nervous system, injured axons maintain the capacity to regenerate spontaneously and provide the possibility for functional recovery post-peripheral nerve injury (Dickson, 2002; Abe and Cavalli, 2008). Peripheral nerve regeneration is accompanied by activation of various complicated molecular pathways and cellular events, which are motivated by differentially expressed genes and significantly changed pathways postinjury. Li et al. (2013) previously profiled global mRNA expression changes in proximal nerve segments, focusing on dynamic changes in the crucial biological processes, and they noted the time-dependent expression of key regulatory genes after sciatic nerve transection. The peripheral nerve injury response leads to the upregulation of various genes required for initiating, guiding, and sustaining axonal growth. Guo et al. (2018) tried to identify specific genes related to the immune response and axon regeneration after peripheral nerve injury. They found six genes, namely, Alcam, Nrp1, Nrp2, Rac1, Creb1, and Runx3, in rat models of sciatic nerve transection, and these genes were found to be partially localized to the axons, especially in the regenerative axons (Guo et al., 2018). Injury to peripheral nerves can activate innate and acquired immune responses. Of note, the success or failure of the regeneration process has been interfered with by the interaction and overlap mechanisms of the nervous and immune systems after nerve injury (Gaudet et al., 2011; Rotshenker, 2011; Xanthos and Sandkühler, 2014). We suggested that regeneration, angiogenesis, and the immune response of the nerve interact and affect each other, and the results of their interaction directly affect the functional recovery of target organs after nerve repair (Yao et al., 2012; Cattin et al., 2015; Guo et al., 2018). Therefore, in this study, we investigated the transcriptome of SD rats in a model of sciatic nerve crush injury induced by clamping and combined the results of our GO and KEGG analyses to identify four genes related to nerve regeneration, angiogenesis, and the immune response: CCR5, Thy1, Notch1, and Sema4A. Among them, only change of Notch1 was basically consistent with the RNA-seq result after injury, which may be responsible for coordinating this process and promote peripheral nerve regeneration.

The DLL4/Notch1/Akt pathway is principally related to angiogenesis because it regulates the proliferation and migration of ECs in both normal conditions and in malignancies (Jones and Li, 2007; Sun et al., 2018; Tian et al., 2018). Inhibition of the Notch1 pathway suppresses ECs proliferation (Sun et al., 2018; Tian et al., 2018). It has also been reported that VEGF triggers DLL4 expression in tip cells to promote migration and activation of stalk cell proliferation via the Notch pathway (Jones and Li, 2007; Hasan et al., 2017; Park et al., 2018). Additionally, components of the Notch1 pathway are highly expressed in arteries but not in veins (Park et al., 2018), where it limits proliferation to prevent an excessively branched vasculature. Notch signaling can activate macrophage mobilization (Bai et al., 2018; Hossain et al., 2018) and promote neural stem and progenitor cell proliferation (Wang et al., 2018). On the one hand, enhancing arterial ECs proliferation and tube formation via the Notch1 pathway is important in angiogenesis during regeneration. On the other hand, the Notch1 pathway modulates artery formation by controlling ECs proliferation. The combination of the effects on immunology and neural cells suggests the importance of Notch1 in neural regeneration after damage. In the current study, we found that Notch1 expression showed significant change at both ends between 4 and 7 days after crush injury. The trend in the Notch1 change was basically consistent with the RNA-seq result after injury. In the proximal ends of injured nerves, Notch1 expression showed significant changes 4 days after the operation and continued to increase at day 7. But in the distal end, 4 days after injury, Notch1 expression was significantly lower than that in the control group, then gradually increased and became significantly higher than that in the control group by day 7 (*P* < 0.01). Furthermore, when we investigated interactions with related genes, we found eight important genes were associated with the Notch signaling pathway, most of which were indispensable in regeneration process of axons or vasculature.

Gene expression of CCR5, Thy1, and Sema4A showed either no changes or changes in the opposite direction, which implied that these three genes didn’t not pass validation. However, it might be because of sites of samples were different between RNA-seq and qPCR analysis.

## Conclusion

After peripheral nerve injury, CCR5, Thy1, Notch1, and Sema4A could be identified by bioinformatics analysis and transcriptional gene screening and found associated with regeneration, angiogenesis, and the immune response. Among them, the Notch1 change was basically consistent with the RNA-seq result after injury, which may be responsible for coordinating this process and promote peripheral nerve regeneration.

## Data Availability Statement

The datasets presented in this study can be found in online repositories. The names of the repository/repositories and accession number(s) can be found below: https://www.ncbi.nlm.nih.gov/, GSE162548.

## Ethics Statement

The animal study was reviewed and approved by Experimental Animal Administration Committee of Sun Yat-sen University.

## Author Contributions

BH, ZZ, and YX designed the study. VP established the animal models. XL, SX, and YZ analysed and interpreted the experimental data. DD and GW were responsible for the bioinformatics analysis. ZZ and VP generated the graphs. YX, ZZ, and BH edited and revised the manuscript. All authors read and approved the final manuscript.

## Conflict of Interest

The authors declare that the research was conducted in the absence of any commercial or financial relationships that could be construed as a potential conflict of interest.

## Publisher’s Note

All claims expressed in this article are solely those of the authors and do not necessarily represent those of their affiliated organizations, or those of the publisher, the editors and the reviewers. Any product that may be evaluated in this article, or claim that may be made by its manufacturer, is not guaranteed or endorsed by the publisher.

## Footnotes

1
http://www.geneontology.org

2
http://www.genome.jp/kegg/

3
http://bioinformatics.psb.ugent.be/webtools/Venn/

4
http://www.cytoscape.org/

5
http://apps.cytoscape.org/apps/GeneMania

## Abbreviations

SD rats: Sprague-Dawley rats
SCs: Schwann cells
NGS: next-generation sequencing
FPKM: fragments per kilobase of transcript per million mapped reads
TMM: trimmed mean of *M* values
FDR: false discovery rate
GO: Gene Ontology
KEGG: Kyoto Encyclopedia of Genes and Genomes
DEmRNAs: differentially expressed mRNAs
FC: fold change
RF: rich factor
DRG: dorsal root ganglion
ECs: endothelial cells
VEGF: vascular endothelial growth factor
ECM: extracellular matrix
ACTM: acellular tissue matrix
Ang: angiopoietin
FGF: fibroblast growth factor
IOD: integrated optical density.
