# Supplementary material for: Interactions Among Nerve Regeneration, Angiogenesis, and the Immune Response Immediately After Sciatic Nerve Crush Injury in Sprague-Dawley Rats
Source: Front Cell Neurosci. 2021 Oct 4;15:717209. doi: 10.3389/fncel.2021.717209 (PMC8522912; doi:10.3389/fncel.2021.717209)
Supplement: Supplementary file 1 [file Data_Sheet_1.docx]

Supp Table 1. Links types related to genes interactive with four common DEmRNAs

| Link Types | Counts | Ratio |
| --- | --- | --- |
| Co-expression | 20 | 0.425532 |
| Co-localization | 2 | 0.042553 |
| Pathway | 7 | 0.148936 |
| Physical Interactions | 4 | 0.085106 |
| Predicted | 14 | 0.297872 |
| Total | 47 |  |

Supp Table 2. Ten top score of genes that are interactive with four common DEmRNAs.

| gene name | score |
| --- | --- |
| Xiap | 0.011594 |
| Fbxw7 | 0.008796 |
| Jag2 | 0.008284 |
| Cul1 | 0.007969 |
| Skp1 | 0.007035 |
| Jag1 | 0.005876 |
| Lfng | 0.004644 |
| Pofut1 | 0.00427 |
| Wdr12 | 0.003947 |
| Ikbkg | 0.003911 |
